# Using Virtual Reality to Bring Restorative Environments to Employees: An Online Pilot Study

**DOI:** 10.3390/ijerph20105797

**Published:** 2023-05-12

**Authors:** Kristin A. Horan, Maria Harrington, Chelsea A. LeNoble, Matthew Mosher, Thomas Pring

**Affiliations:** 1 Department of Psychological Science, Kennesaw State University, Kennesaw, GA 30144, USA; 2 Department of Games and Interactive Media, University of Central Florida, Orlando, FL 32801, USA; 3 Department of Psychology, University of Central Florida, 4000 Central Florida Blvd., Orlando, FL 32816, USA

**Keywords:** work recovery, restorative environments, physical activity, nature contact, virtual reality

## Abstract

Employees face many demands throughout the workday. Participating in activities can help employees recover from the pressures of work, and physical activity and time spent in nature are among the most beneficial. Simulations of nature offer some of the benefits of actual contact with nature and can address some of the barriers to exercising outdoors that some employees may face. In this pilot study, we examine the influence of physical activity and virtual or actual nature contact on affect, boredom, and satisfaction when experienced during a break from a demanding work task. Twenty-five employed adults participated in an online study in which they completed a problem-solving task, completed a twenty-minute break, and then completed another session of the problem-solving task. During the break, participants were randomized to either a control condition, a physical activity and low-fidelity virtual nature contact condition, a physical activity and high-fidelity virtual nature contact condition, or a physical activity and actual nature contact condition. An examination of the means of affect, boredom, and satisfaction before, during, and after the break revealed that those in high-fidelity virtual nature and actual nature contact conditions seemed to report more positive well-being during the break. The results highlight that to help employees recover from work demands, it could be important to take breaks, be physically active, and have contact with nature, which should be simulated in high fidelity if actual nature contact cannot be achieved.

## 1. Introduction

Work is associated with many demands; therefore, it is important that employees can restore resources that have been depleted through the course of the workday [1]. Employees and organizations face adverse health- and performance-related consequences if employees are not able to restore their depleted resources [2]. Even in the context of a short work break, spending time performing certain activities, such as physical activity [3] or experiencing a nature environment (here termed nature contact) [4], contributes to the restoration of depleted resources. Despite these benefits, there can be barriers to encouraging time spent in nature or time spent performing physical activity, such as the absence of safe or readily accessible natural environments. More research and advances in traditional restorative environment paradigms are needed to reduce these barriers to participation in physical activity and nature contact. Recent advances in virtual reality present opportunities for employees to experience simulated nature contact [5], which could address some barriers to physical activity and traditional nature contact. However, the extent to which virtual nature contact replicates the benefits of real-life experiences is not fully understood. The present study is a pilot examination of the influence of physical activity and virtual nature contact as a break during a problem-solving task and their effect on the workplace well-being outcomes of affect, boredom, and task satisfaction.

### 1.1. Work Demands, Restorative Activities, and Well-Being

Employees are exposed to many demands as they work, including demands related to time, social interactions, and the physical environment [6]. Due to the psychological and physiological cost of increased and sustained effort to meet these demands, repeated or chronic exposure to such demands depletes employee resources and jeopardizes performance and well-being [6,7]. For example, according to the episodic process model [7], periods of goal-directed behavior, such as the sustained effort needed to perform a work task, will require cognitive and regulatory resources. The episodic process model has been applied to examine within-person fluctuations in both performance [7] and well-being [8]. Cognitive and regulatory resources are depleted throughout the course of episodes of goal-directed behavior but can be replenished or recovered [7,9].

Employees can participate in activities to promote recovery, or the restoration of depleted resources, even during short breaks [10,11]. Employees can participate in a variety of behaviors or activities during short breaks, such as taking a smoking break, taking a coffee break, or going for a short walk, each with varying implications for restoration and long-term health [3]. Research focused on the effectiveness of various recovery activities performed during work breaks has identified that exercise and nature contact are among the most effective recovery activities [12,13]. Participation in these activities is associated with optimal psychological states indicative of a return to pre-demand levels of well-being, such as a more positive mood (i.e., affect), enhanced attention, enhanced attitudinal perceptions (i.e., satisfaction), and reduced stress [11,14].

This restorative process has been viewed from several theoretical traditions. For example, according to stress reduction theory [15], nature contact promotes restoration through positive aesthetic and affective reactions towards nature, promoting a calming state and a reduction in stress [16]. When viewing nature contact through the lens of attention restoration theory [17], nature contact promotes the restoration of depleted attention resources by providing an opportunity to “get away” from demands, experience an expansive state, promote intrinsically motivated activities, and experience stimuli that involuntarily direct and hold our attention towards nature [18]. According to the episodic process model, recovery opportunities such as nature contact promote positive affective processes and replenish cognitive and self-regulatory resources that were depleted during previous episodes of performance [7]. Together, these models and associated empirical studies suggest that recovery activities such as physical activity and nature contact promote the restoration of the resources that an employee depletes during work episodes.

### 1.2. Virtual Reality and Restorative Environments

Despite the many benefits of exercise and nature contact during work breaks, employees can face challenges in implementing these restorative practices during their workday. For example, it might not be possible for employees to leave their work site to take a break outdoors. They may lack access to safe outdoor spaces, may be located in geographic locations that frequently experience inclement weather, or may desire to avoid certain aspects of physical activity or time outdoors, such as exposure to sunlight or heat. There are many ways in which a workplace can be designed to feature more of a connection to nature, termed biophilic work design [19], which vary in the depth and scope of contact with nature. For example, Klotz and Bolino [19] list “outdoor breaks, outdoors brought indoors, outdoors via physical barriers, and representations of nature” as possible elements of biophilic work design that could promote restoration. It is encouraging that some research has demonstrated evidence of the restorative benefits achieved within the context of a built environment and simulated environments. In their review of recovery, Sonnentag and colleagues summarize their research finding that recovery experiences can be promoted in urban spaces designed to feature “green” elements by looking at pictures or videos of nature, or by listening to recorded nature sounds [11]. Thus, for employees who face barriers related to spending time outdoors at work, it is encouraging that recovery can be achieved in built and simulated environments.

However, it is important to acknowledge that some environments are more suitable for recovery than others [11]. An environment’s restorative capacities vary according to the type of exposure, duration of exposure, frequency, and spatial scale [20]. Just as actual outdoor environments can vary in their restorative capacity, it stands to reason that simulations of outdoor nature environments also vary in their restorative capacity. This variability may be a function of the fidelity of the simulation, or the extent to which the simulation accurately replicates the real-world nature experience. Advances in technology-generated imagery, such as virtual reality, have enabled the creation of simulated nature environments that better mimic the visual and auditory features of outdoor nature environments [21]. These visual features (e.g., green, blue, and brown colors) and auditory features (e.g., trickling water or the sound of a bird’s song) have been called the “cues and clues” that trigger the recovery experience and prompt improved states of health and well-being [21]. Using virtual reality, nature simulations can present cues that have higher fidelity, prompting the user to feel more immersed and present in the simulation [22]. Research on virtual reality simulations of nature environments shows promise in promoting recovery through promoting relaxation, reducing stress, restoring attention, and improving cognitive performance [23,24].

### 1.3. The Present Study

In light of empirical evidence of the benefits of physical activity and nature contact to restore resources depleted through work demands, and considering the opportunities afforded by virtual reality to present high-fidelity simulations of a traditional nature context, this pilot study aims to examine the effect of physical activity and nature contact on state well-being during a break from a demanding task. We will seek to answer the following research question: To what extent will virtual nature contact during a break from a demanding task influence patterns among psychological states (affect, satisfaction, and boredom) compared to real or no nature contact?

## 2. Materials and Methods

### 2.1. Context of the Pilot Study

This study is a pilot study that was performed as part of a larger program of research on physical activity, including active workstations that permit sitting, standing, cycling, or walking during a task or break, and the effect of exposure to a photorealistic, immersive virtual nature environment. The research team designed a study with a larger sample size, incorporating in-person exposure to the virtual nature environment, the use of the active workstation, and the measurement of both self-reported and physiological indicators of well-being. However, prior to the initiation of recruitment for this study, all in-person data collection was suspended due to the COVID-19 pandemic. In response, the research team adapted the study design by developing and implementing a pilot study that could be conducted on a smaller scale, using only online data collection techniques.

### 2.2. Participants

Participants were all employed adults. The need to adapt the pilot study so that it could be conducted completely online meant that participants would need to have access to a virtual reality-compatible computer that could run the virtual nature model in their own homes. In order to reach employed adults who were likely to own this type of equipment, the researchers recruited subjects for this study by sending an email to employers who develop online games. The employers were provided with a recruitment email to distribute to employees, and interested employees signed up through email. To be eligible to participate, individuals needed to be employed at least part-time, be able to walk comfortably for at least 20 min, have access to a safe space to walk outdoors, have a virtual reality-compatible computer at home or a dedicated graphics card for running Unreal Engine, be at least 18 years of age, have normal or corrected-to-normal vision and hearing, and have at least one hour during or immediately after their work day to participate. Participating immediately after their morning or afternoon work session ensured that they started the experiment with a level of depletion from work demands that is typical of a normal workday and that they would have not already participated in some restorative activity prior to the study. The final sample of analyzed data included twenty-five employed adults (52% male; 44% White/Caucasian).

### 2.3. Procedures

Once the prospective participants indicated their interest in the study, a research assistant corresponded with them through email to verify their eligibility and to schedule a convenient time for a one-hour virtual Zoom meeting (Zoom Video Communications, San Jose, CA, USA). All virtual sessions were recorded. The research assistant also gave the participants instructions prior to their experimental session, which indicated that participants should schedule their session immediately after a four-hour work period, avoid taking a break during the last hour of their work period, and wear comfortable clothes and shoes for participation. Prior to the experimental session, each participant completed a baseline demographic survey.

**Figure 1:**
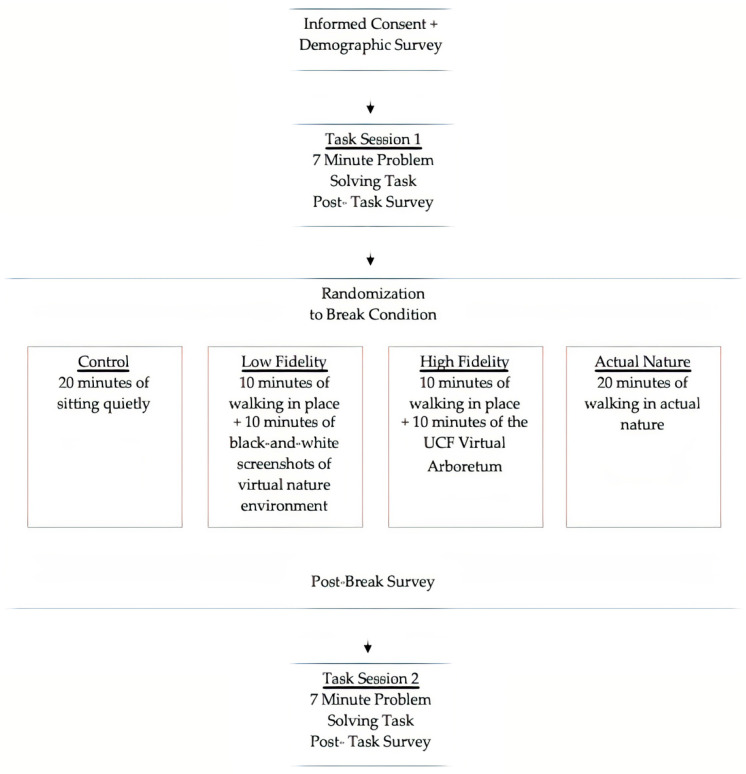
The study protocol.

The study protocol is summarized in Figure 1. A research assistant began each session by explaining the research tasks and obtaining informed consent. Participants then completed a seven-minute problem-solving task, designed to simulate the demands of a work task. Specifically, participants were provided with an image of the nine-dot problem through the chat feature of the Zoom session. The nine-dot problem is an insight problem-solving task [25], meaning that typically participants will solve the problem with a sudden realization of the solution, or an “aha moment,” if they reach the solution at all. The nine-dot problem has a low base rate of successful completion [26] and therefore represents a demanding task similar to that which might be experienced at work. Participants opened the image on their computer, shared their screen, and demonstrated potential solutions using Paint or a similar drawing program, allowing the research assistant to verify whether their solution was correct. The research assistant then administered a three-minute survey that measured state well-being felt during the first problem-solving task session.

**Figure 2:**
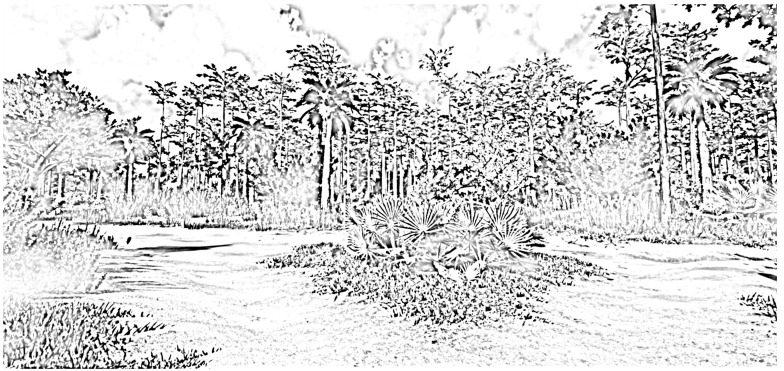
Screenshot from the low-fidelity virtual nature condition. Botanically correct representations of plants native to this region, such as the saw palmetto (*Serenoa repens*) at the center of the screenshot, comprised the imagery.

**Figure 3:**
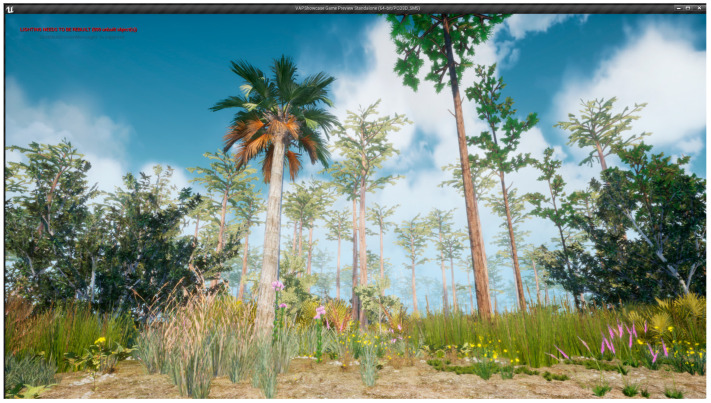
Screenshot of the high-fidelity virtual nature conditions of the Virtual UCF Arboretum (The Harrington Lab at University of Central Florida, Orlando, FL, USA). Native grasses such as the chalky bluestem (*Andropogon virginicus* var. *glaucus*) and wildflowers such as blazing stars (*Liatris* spp.) can be seen in bloom in this virtual space, just as they would be in the real world.

Participants were then given a twenty-minute work break that comprised the experimental portion of the pilot study. The research assistant referred to a spreadsheet that contained the participant’s ID number, along with a randomly generated number that corresponded to an experimental condition. The first condition was the control condition, in which the participant had no physical activity and no nature contact. They were instructed to sit quietly for twenty minutes. The second condition involved ten minutes of light physical activity (i.e., walking in place) and ten minutes of low-fidelity virtual nature contact (i.e., watching a slideshow on their desktop computer with black-and-white screenshots of the virtual reality nature environment; see Figure 2). The third condition involved ten minutes of light physical activity (i.e., walking in place) and ten minutes of high-fidelity virtual nature contact (i.e., navigating the virtual nature environment on their desktop computer; see Figure 3). The fourth and final condition involved 20 min of physical activity in a real nature environment. Specifically, participants randomly assigned to this condition were instructed to go outside and take a walk. They were told to set a timer on their phone for 10 min, which would give them a cue to turn around and head back to their home. For all conditions involving physical activity, the participant was instructed that they may choose their own pace of walking. For the high fidelity and real nature conditions, participants had control over their path, meaning that they could navigate anywhere they would like in the virtual reality nature scene or on their outdoor walk. At the conclusion of their 20-minute break, all participants were given a three-minute survey assessing state well-being felt during their break.

Next, participants were given another seven minutes to return to the nine-dot problem, which simulated the experience of returning to a mentally demanding work task following a break. After the final problem-solving task, participants were given three minutes to complete a final measure of state well-being. Participants were thanked for their time, and the experimental session concluded. At this point, the Zoom recording was stopped, and the Zoom meeting was ended. Participants received a USD 20 electronic Amazon gift card as a participation incentive.

#### The Virtual UCF Arboretum

For conditions involving virtual nature contact, participants were exposed to the Virtual UCF Arboretum (The Harrington Lab at University of Central Florida, Orlando, FL, USA) [27]. A screenshot is provided in Figure 2. The Virtual UCF Arboretum is a 100-hectare (247-acre) virtual model that was constructed using the Epic Games Unreal Engine, (Epic Games, Cary, NC, USA), which can utilize both virtual reality and augmented reality applications. The model contains realistic, botanically correct 3D models of plants constructed using actual plant population data, along with accurate representations of insects, amphibians, and birds, accompanied by ambient acoustics. A YouTube video demonstrating the Virtual UCF Arboretum (S1) and a software download link (S2) are provided in Supplementary Materials. 

### 2.4. Measures

The following measures were selected because they have been used in previous studies to indicate task-related well-being, including use in studies focused on the influence of active workstations on task performance and state well-being [28]. They are also regarded as psychological states indicative of the replenishment or recovery of resources depleted through demanding work tasks (i.e., the presence of positive psychological states such as positive affect and satisfaction and the absence of negative psychological states such as negative affect and boredom) [11]. Participants completed each measure during the survey administered immediately after the first problem-solving task, immediately after their break, and immediately after the second problem-solving task.

#### 2.4.1. Affect

State affect was measured using the positive and negative affect schedule [29]. This 20-item scale asks participants to state the level of affect that they felt using words of positive and negative valence, such as “interested” and “alert” for the positive affect subscale and “distressed” and “upset” for the negative affect subscale. Participants responded on a scale from 1 (“very slightly or not at all”) to 5 (“extremely”). Previous research has demonstrated that recovery experiences can contribute to positive and negative affect in distinct ways [11], warranting the inclusion of both positive and negative affect measures in this study. The internal consistencies for the positive affect subscale reached satisfactory levels (Task Session 1 α = 0.78, Break α = 0.83, Task Session 2 α = 0.79). The internal consistencies for the negative affect subscale were generally acceptable, falling slightly below satisfactory levels (Task Session 1 α = 0.69, Break α = 0.69, Task Session 2 α = 0.68).

#### 2.4.2. Boredom

State boredom was measured using four items from the job boredom scale [30]. The scale was adapted to increase contextualization and to refer to boredom during a laboratory task [28], as opposed to boredom with one’s job in general. An example item includes, “During this task/break, time went by very slowly”. Participants were asked to rate the extent to which they agreed with the statement and responded on a scale from 1 (“strongly disagree”) to 5 (“strongly agree”). The internal consistencies reached strong to satisfactory levels (Task Session 1 α = 0.93, Break α = 0.87, Task Session 2 α = 0.74).

#### 2.4.3. Task Satisfaction

Satisfaction with a task was measured using three items from the Michigan organizational assessment questionnaire [31] that were adapted to increase contextualization and refer to satisfaction with a laboratory task [28], rather than satisfaction with one’s job in general. An example item includes, “In general, I liked working on this task.” Participants were asked to rate the extent to which they agreed with the statement and responded on a scale from 1 (“strongly disagree”) to 5 (“strongly agree”). The internal consistencies reached satisfactory levels (Task Session 1 α = 0.85, Break α = 0.77, Task Session 2 α = 0.84).

## 3. Results

**Figure 4:**
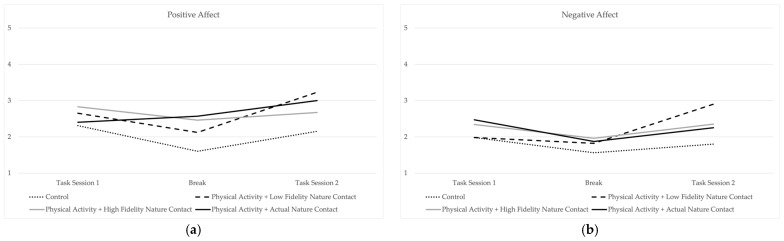
Mean patterns for (**a**) positive affect and (**b**) negative affect.

**Figure 5:**
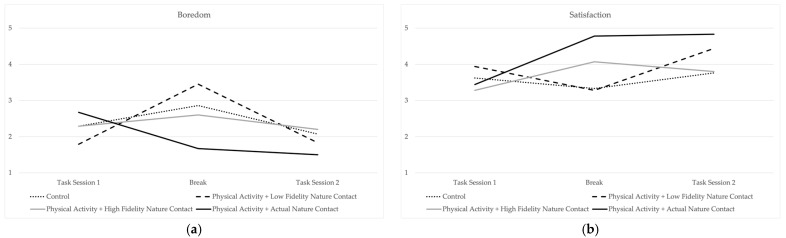
Mean patterns for (**a**) boredom and (**b**) task satisfaction.

The means of state well-being measures for the first task, the break, and the second task, broken down according to experimental conditions, are displayed in Table 1. Means for positive and negative affect are visually displayed in Figure 4 and means for boredom and task satisfaction are visually displayed in Figure 5. The small sample size associated with this pilot study resulted in a small number of participants in each condition, precluding the use of statistical significance testing. However, a visual inspection of the patterns of the means does provide some general insights, particularly when examining state well-being during the break itself. The control condition, which was associated with no physical activity and no nature contact, tended to have the lowest levels of positive states (i.e., positive affect, satisfaction). Levels of state well-being tended to be more positive during the break for those in the high-fidelity nature contact condition and in the actual nature contact condition. Although the means of state well-being during the second task session generally displayed a pattern of returning to pre-break levels, there were some cases in which post-break patterns illustrated the sustained benefits of recovery activity. In some cases, means in these conditions displayed trajectories illustrative of the sustained benefits of restorative environments. For example, positive affect increased both in the break and in the second task session for those in the actual nature conditions. Satisfaction increased during the break for those who walked in actual nature and those who navigated the high-fidelity nature environment. Although satisfaction did decrease in the second task session for those in the high-fidelity condition, their second task session satisfaction level was still higher than in the first task session. For those in the actual nature condition, the levels of satisfaction remained consistently high in the second task session.

## 4. Discussion

This pilot study leverages the literature on the benefits of physical activity and nature contact during short periods of recovery from work demands, while incorporating a high-fidelity virtual nature simulation. Trends in the patterns of means across conditions and task sessions/breaks, particularly when looking at well-being during the breaks, suggested a potential benefit of nature contact during work breaks. Means for state well-being for participants in the high-fidelity virtual nature condition and the actual nature condition, both of which contained more of the “cues and clues” thought to prompt recovery states in restorative environments [21], trended higher for levels of positive states, with lower levels of negative states during the break. These results are consistent with previous research documenting the benefits of virtual nature exposure [23], including in the context of recovery from work demands [24]. Additionally, our findings that actual and immersive virtual reality simulations of nature can promote similar levels of restoration are in line with previous findings [23].

This study is strengthened by the inclusion of an innovative, novel form of nature contact: a high-fidelity virtual nature environment constructed as a replica of real natural lands. The inclusion of this virtual model aligns with calls for more research that leverages technology to aid in the process of recovery from work demands [13] since the inclusion of virtual nature contact as an option for a recovery setting in the workplace could help overcome some of the obstacles associated with outdoor nature contact. The findings and generalizability of this pilot study are limited by several factors. First, the study is limited by the small sample size. The adaptation of the study to the context of COVID-19, in which participation took place virtually instead of in a laboratory environment, inherently limited the population of eligible participants who owned virtual-reality-compatible computers in their homes. This negatively impacted the pace of participant recruitment and the sample size itself. In addition to the small overall sample size, it should also be noted there is an unequal number of participants in each condition. This occurred due to the nature of the random number generator, as our random number generator assigned a participant to a condition without taking the frequency of previous assignments into account (i.e., the recommendations are truly random) because it is assumed that the random nature of the generator will result in approximately equal cell sizes as the total sample size grows. As we were originally pursuing a larger sample size, we chose to assign participants based on the truly random number suggested by the generator and expected that cells would have reached more equivalent sample sizes at a final higher overall sample size. When recruitment options were exhausted due to the strict eligibility criteria of the COVID-19-related adaptation, cell sizes did not include an equal number of participants. Due to the limitations associated with the overall sample size and the cell size of each condition, generalizability cannot be assumed, and these findings should be interpreted as preliminary evidence. Second, the study is also limited by the fact that the experimental protocol did not establish a comprehensive baseline of work demands or state well-being for participants. That is, although our pre-study instructions aimed to minimize the differences in opportunities for restoration across participants by instructing them to work for a four-hour window and to avoid breaks in the last hour prior to the experiment, some participants may have experienced more demanding workdays, may differ in their levels of trait well-being, or started the experiment at different rates of state well-being.

Despite the limitations associated with this pilot study, these initial findings should encourage researchers to further investigate the impact of nature contact (real or virtual) on important workplace well-being outcomes. In future research that builds upon this pilot study, these results can be replicated with higher sample sizes and equal sample sizes within each condition. Future studies based on this pilot can pursue higher levels of experimental or statistical control of the baseline levels of trait well-being, pre-experimental state well-being, and pre-experimental work demands. This study can be extended by incorporating other forms of technology, such as an active workstation, to increase the physical activity fidelity of the study design. In addition, should future research replicate these findings, additional work can be dedicated to understanding the underlying mechanisms that explain how and why nature-based work breaks convey positive effects. Identifying such explanatory factors could assist virtual reality developers in determining where to focus their fidelity enhancements. Such examinations of mediators and the underlying mechanisms could also aid researchers in identifying the most pertinent theoretical lens and associated operationalizations of variables for future studies investigating physical activity and nature contact from a work recovery perspective.

## 5. Conclusions

In line with previous research on the benefits of physical activity and nature contact, even simulated nature contact, this pilot study found that state well-being tended to be more positive during a break from a demanding work task in participants who engaged in physical activity and explored a high-fidelity or actual nature environment. In some cases, the sustained state well-being endured beyond the respite, such as in post-break task satisfaction. The results highlight the importance of policies, practices, and spaces in workplaces that support employees taking breaks, being physically active, and engaging with actual or simulated nature. The results also remind scholars and practitioners that when creating spaces that encourage restoration, we must include high-fidelity elements such as colors and sounds that promote recovery.

## Figures and Tables

**Table 1:** Descriptive statistics for measured variables by condition.

	Control (N = 7)	Physical Activity +	Physical Activity +	Physical Activity +
		Low-Fidelity Nature Contact	High-Fidelity Nature Contact	Actual Nature Contact
		(N = 6)	(N = 9)	(N = 3)
Positive Affect	TS1: 2.31 (0.62)	TS1: 2.65 (0.77)	TS1: 2.83 (0.85)	TS1: 2.40 (0.62)
	B: 1.60 (0.52)	B: 2.12 (0.62)	B: 2.46 (0.67)	B: 2.57 (0.71)
	TS2: 2.15 (0.64)	TS2: 3.23 (0.42)	TS2: 2.67 (0.85)	TS2: 3.00 (0.57)
Negative Affect	TS1: 1.98 (0.46)	TS1: 1.98 (0.54)	TS1: 2.34 (0.59)	TS1: 2.47 (1.00)
	B: 1.56 (0.42)	B: 1.82 (0.43)	B: 1.96 (0.76)	B: 1.87 (0.32)
	TS2: 1.80 (0.56)	TS2: 2.90 (0.26)	TS2: 2.35 (0.52)	TS2: 2.25 (0.49)
Boredom	TS1: 2.29 (1.29)	TS1: 1.79 (0.87)	TS1: 2.29 (1.10)	TS1: 2.67 (1.59)
	B: 2.86 (0.98)	B: 3.45 (1.30)	B: 2.60 (1.08)	B: 1.67 (0.52)
	TS2: 2.07 (0.59)	TS2: 1.83 (0.72)	TS2: 2.20 (0.48)	TS2: 1.50 (0.71)
Satisfaction	TS1: 3.62 (0.62)	TS1: 3.94 (1.02)	TS1: 3.28 (1.39)	TS1: 3.44 (1.58)
	B: 3.33 (0.86)	B: 3.28 (1.18)	B: 4.07 (0.60)	B: 4.78 (0.38)
	TS2: 3.76 (0.57)	TS2: 4.44 (0.96)	TS2: 3.80 (0.77)	TS2: 4.83 (0.24)

Note: Means are presented, followed by standard deviations in parentheses. TS1 = Task Session 1, B = Break, TS2 = Task Session 2.

## Data Availability

Data is available from the corresponding author upon request.

## Supplementary Materials

The following supporting information can be downloaded at: https://www.mdpi.com/article/10.3390/ijerph20105797/s1, Video S1: https://www.youtube.com/@mariaharrington2565/videos (accessed on 14 February 2023); Software Download S2: The Virtual UCF Arboretum, software download: https://the-harrington-lab.itch.io/the-virtual-ucf-arboretum (accessed on 14 February 2023).
